# Gasohol Quality Control for Real Time Applications by Means of a Multimode Interference Fiber Sensor

**DOI:** 10.3390/s140917817

**Published:** 2014-09-25

**Authors:** Adolfo J. Rodríguez Rodríguez, Oscar Baldovino-Pantaleón, Rene F. Domínguez Cruz, Carlos R. Zamarreño, Ignacio R. Matías, Daniel A. May-Arrioja

**Affiliations:** 1 Fiber and Integrated Optics Laboratory, Electronics Engineering Department, UAM Reynosa Rodhe, Universidad Autónoma de Tamaulipas, Carr. Reynosa-San Fernando S/N, Reynosa, Tamaulipas 88779, Mexico; E-Mails: arodriguez@uat.edu.mx (A.J.R.R.); obaldovino@gmail.com (O.B.-P.); rfdominguez@uat.edu.mx (R.F.D.C.); 2 Departamento de Ingeniería Eléctrica y Electrónica, Universidad Pública de Navarra, Edif. Los Tejos, Campus Arrosadia, Pamplona 31006, Spain; E-Mails: carlos.ruiz@unavarra.es (C.R.Z.); natxo@unavarra.es (I.R.M.)

**Keywords:** optical fiber sensor, multimode interference, MMI, gasohol, gasoline, ethanol

## Abstract

In this work we demonstrate efficient quality control of a variety of gasoline and ethanol (gasohol) blends using a multimode interference (MMI) fiber sensor. The operational principle relies on the fact that the addition of ethanol to the gasohol blend reduces the refractive index (RI) of the gasoline. Since MMI sensors are capable of detecting small RI changes, the ethanol content of the gasohol blend is easily determined by tracking the MMI peak wavelength response. Gasohol blends with ethanol contents ranging from 0% to 50% has been clearly identified using this device, which provides a linear response with a maximum sensitivity of 0.270 nm/% EtOH. The sensor can also distinguish when water incorporated in the blend has exceeded the maximum volume tolerated by the gasohol blend, which is responsible for phase separation of the ethanol and gasoline and could cause serious engine failures. Since the MMI sensor is straightforward to fabricate and does not require any special coating it is a cost effective solution for real time and *in-situ* monitoring of the quality of gasohol blends.

## 1. Introduction

In the last two decades there has been a growing interest in the development of renewable fuels that might replace or reduce the use of gasoline. This has been motivated by the fact that petroleum is not a renewable source, and the neverending increase in production costs, as well as pollution problems related to gasoline use in the majority of automotive vehicles. Among the different approaches to develop renewable fuels, ethanol has attracted significant interest because it can be used either as a replacement or an additive for gasoline. The mixture of gasoline and ethanol is known as gasohol. The main advantage when using gasohol is that the higher oxygen content of ethanol allows for greater fuel economy and reduction of contaminant emissions [1–3]. As a result, gasohol with different gasoline and ethanol mixtures are currently used in different countries. Nevertheless, there are some issues that need to be taken into account when using gasohol in small engines. Due to the hygroscopic and miscibility properties of ethanol, water can be absorbed from atmosphere and it dilutes the ethanol. The main issue here is that if we have a higher fraction of water than what can be contained by the gasohol mixture, phase separation of the gasohol mixture will occur. This produces abnormal combustion and leads to engine knocking that can potentially damage the engines. On the other hand, ethanol is known to increase the corrosion of the engine and fuel system materials due to soluble contaminants such as chloride ions. However, it has been shown that the addition of water can help to prevent corrosion as well, but the fraction of water has to be carefully controlled to avoid phase separation. We should also mention that in some countries, Brazil for example, the use of gasohol blends with higher percentages of ethanol is legal. Nevertheless, since ethanol has lower price (about 60% less) than gasoline, a common malpractice is to increase ethanol concentration in the mixture that is sold to car owners. Therefore, the gasohol blend and their water content should be monitored not only when the blend is distributed, but also in real time when gasohol is being used.

There are a few techniques to detect alterations in gasoline either due to adulteration or ethanol incorporation, such as the incorporation of chemiresistors for ethanol detection in hydrocarbons [4], piezoresonance elements [5], and the use of sensor arrays based on mass and capacitance transducers [6]. However, for security reasons, it is highly desirable that such sensors avoid electrical signals due to the contact with flammable or explosive substances. There are other options based in selective colorimetric indicator films called Wetting In Color Kit (WICK) [7], but chemical interference presents a significant inconvenient as a sensing element. Optical fiber sensors (OFS) are ideal for this task because they do not require electrical signals to operate. In addition, OFS are compact, immune to electromagnetic interference, exhibit high sensitivity, with good portability and low cost. The majority of the OFS involved with fuel detection has been focused on hydrocarbon leak detection [8–11]. There are few reports dealing with the detection of the percentage of ethanol and other contaminants in gasoline [12–14]. A particular technique relies on the extraction of analyte molecules into a hydrophobic silicone cladding that covers an optical fiber and the measurement is performed via absorption changes of the evanescent field [12]. The main drawback is that the response time is large and the fiber requires additional preparation. Other reports take advantage of the ability of long period gratings (LPG) to measure the refractive index (RI) of liquids as a way to detect mixtures of ethanol and gasoline [13,14]. The only drawback in this case, is the need to inscribe the LPG which requires complex equipment and could impact the final cost of the sensor. A device that has attracted a great deal of interest on the development of fiber sensors is the one based on multimode interference (MMI) effects. The key advantages of MMI fiber sensors are that they are quite simple to fabricate and relatively inexpensive. Therefore, fiber sensors based on MMI structures have been developed to measure different variables such as temperature, curvature, vibration, liquid level, as well as MMI refractometers [15–21].

In this paper we demonstrate efficient quality control of a variety of gasohol blends using MMI fiber sensors. As we previously explained a particular gasohol blend is defined by the volume concentration of ethanol incorporated into the blend. Considering that ethanol has a smaller refractive index than gasoline, we expect that gasohol blends with higher ethanol content will exhibit a smaller RI than gasoline. Since MMI sensors are capable of detecting small RI changes, accurate control of gasohol blends is realized in a simple way. Additionally the sensor is capable of detecting when water incorporated in the blend has exceeded the maximum volume tolerated by the gasohol blend, which is responsible for phase separation of the ethanol and gasoline. We should highlight that since the sensor does not require any particular coating and its fabrication is rather simple and inexpensive.

## 2. Principle of Operation

**Figure 1.**
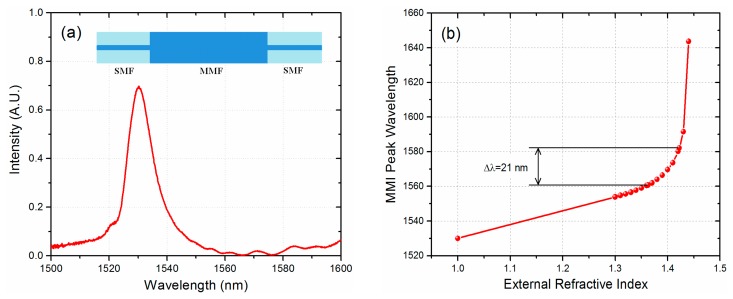
(**a**) Spectral response of an MMI device (Inset: schematic of a MMI device) and (**b**) MMI peak wavelength response as a function of the RI of the external media.

A MMI structure is fabricated by splicing a multimode fiber (MMF) section between two single mode fibers (SMF). Light launched into one of the SMF will reach the MMF and, as the light propagates through the MMF, we observe the formation of periodic images of the input field along the MMF at specific locations [16]. Therefore, if the MMF is cleaved to a particular length, which coincides with the position where an image is formed, light with a specific wavelength will be coupled to the SMF output and transmitted through the MMI device. Any other wavelength value that deviates from the design wavelength will form its image before or after the MMF-SMF interface, and the light coupled to the output SMF will be attenuated as shown in Figure 1a. The relation that defines the transmitted MMI peak wavelength is well known and is given by [19]:

(1) $$\lambda =p\left(\frac{n_{\text{MMF}}D_{\text{MMF}}^{2}}{L}\right),\;\text{with}\;p=0,\;1,\;2\;....,$$

where *D_MMF_* and *n_MMF_* are the effective diameter and effective refractive index (RI) of the fundamental mode of the MMF respectively, *λ* is the free-space wavelength, and *L* is the length of the MMF. As shown in Equation (1), the peak wavelength can be shifted when the effective RI and diameter are modified, which can be achieved via the evanescent field of the propagating modes. In order to allow the modes to interact with the surrounding media we use a MMF known as No-Core fiber, which is a MMF without cladding (*i.e.*, air is the cladding). Therefore, when the MMI device is immersed in a liquid, such as gasoline, ethanol, or gasohol in our case, the index contrast between core and liquid cladding will be reduced which increases the effective diameter and RI of the fundamental mode. The net result is that the MMI peak wavelength will be shifted to longer wavelengths as the RI of the liquid is increased. Since there is a significant RI difference between gasoline and ethanol, such effect can be used to evaluate the quality of gasohol mixtures.

In order for the MMI sensor to discriminate the gasohol mixtures the spectral separation between the transmitted MMI peaks when the No-Core fiber is surrounded by ethanol and gasoline has to be clearly identified. Using a finite element method software (COMSOL Multiphysics) we can obtain the effective diameter and RI of the fundamental mode, when the MMI is immersed in both liquids, and these values are then used in Equation (1) to obtain the transmitted MMI peak wavelength for a fixed MMF length. The No-Core MMF parameters used in the simulations are a core RI of *n* = 1.444 and a diameter of 125 μm. We consider the RI at 1550 nm of ethanol and gasoline as *n* = 1.3622 and *n* = 1.4223 respectively [14]. The length of the No-Core fiber was taken as 58.98 mm, which corresponds to a MMI peak wavelength of 1530 nm with air as the cladding. We also included other RI values to obtain a better curve. As shown in Figure 1b in the case of ethanol the transmitted peak is located at 1560.75 nm whilst for gasoline is located at 1582.08 nm. The peak-to-peak difference of 21 nm should be enough to identify different gasohol mixtures whose MMI peak wavelength will fall within this range. Nevertheless, as will be shown later, we can slightly increase the sensitivity by reducing the diameter of the No-Core fiber.

## 3. Experimental Design

**Figure 2.**
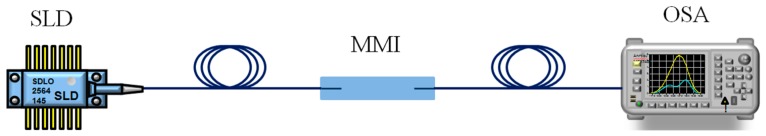
Experimental setup for gasohol measurements.

The No-Core fiber used in our experiments was acquired from the company Prime Optical Fiber Corporation (Miao-Li County, Taiwan). The MMI sensor was fabricated by first splicing the SMF to one end of the No-Core MMF. Using a microscope and a micrometer stage we align the splicing point with the edge of the cleaver knife, and the fiber is then moved away a distance of 59.58 mm. The No-Core MMF length is slightly larger as estimated from Equation (1) for peak wavelength transmission at 1530 nm and air as the surrounding medium, which could be related to slight variations of the RI and diameter of the NO-Core MMF. The No-Core MMF is finally cleaved and spliced to another SMF, and at this stage the sensor is ready for testing. We should highlight that the surface of the No-Core fiber should be free of any polymer that could interfere with the measurements. Therefore, after fabrication, the MMI device was cleaned using a sulfuric acid solution (1M) to remove any residual polymer. The experimental setup for testing the MMI gasohol sensors is quite simple and is shown in Figure 2. A superluminescent diode (SLED) with a wavelength range from 1465 to 1650 nm was used as the broadband optical source, which is connected to the input SMF using FC/PC connectors. The transmitted spectrum through the MMI sensor is then measured using an optical spectrum analyzer (OSA).

**Figure 3.**
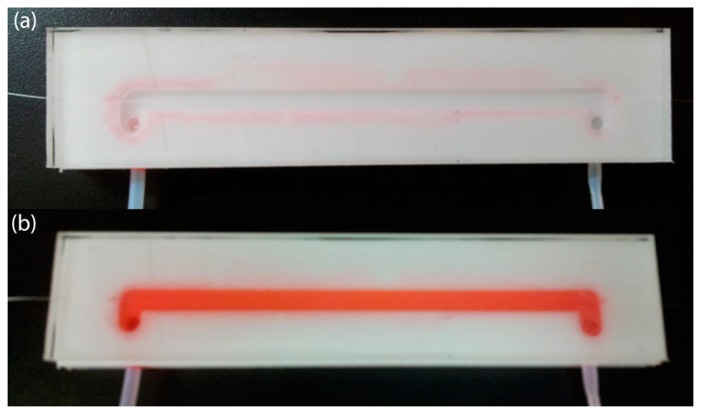
MMI sensor fixed into the Delrin channel with glass cover (**a**) without gasohol and (**b**) with gasohol. Red dye is added to the gasohol solution to highlight the gasohol in the channel.

The MMI structure was fixed into a channel engraved in a Delrin plate with integrated liquid inlet and outlet channels. As shown in Figure 3a glass cover was glued on top of the Delrin plate in order to seal the channel. The channel without and with gasohol are shown in Figure 3a,b respectively, with red dye added to the gasohol blend to facilitate visualization inside the channel.

During the measurements we monitored the temperature (∼23 °C) and humidity (∼26%), and small variations of less than 4% were observed during the experiments. Such small variation does not significantly alter the response of the sensor. It is important to mention that in Mexico we have two different kinds of gasoline with 87 and 92 octane, named G87 and G92 respectively, and both are free of ethanol. Therefore, in order to obtain different gasohol blends, we prepared different mixtures of G87 diluted with anhydrous ethanol (AE) as shown in Table 1. The mixtures were selected according to the different gasohol blends that are commonly used in several countries [1–3]. We should also highlight that AE was used to guarantee that the gasohol blends do not contain or will absorb water. Although the results reported here were performed using the G87 type, similar results should be obtained with the G92 type. We believe that similar results should be obtained for other types of gasoline used worldwide.

## 4. Experimental Results

**Figure 4.**
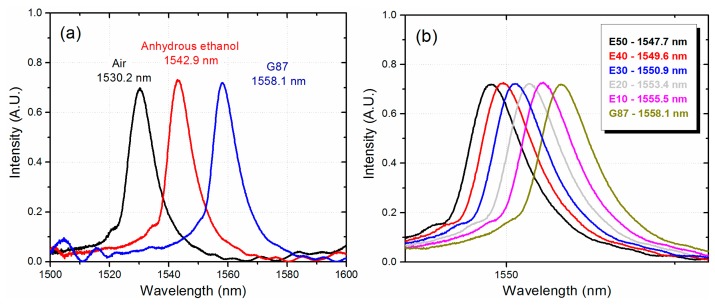
(**a**) Spectral response of the MMI sensor for anhydrous ethanol and G87 gasoline; (**b**) Gasohol blends from Table 1 (MMF diameter of 125 μm).

We first measured the spectral response of the MMI sensor when is covered with AE and G87 gasoline. As shown in Figure 4a, we have a separation of 15.2 nm between the transmitted peak wavelengths which is smaller than the expected values obtained from Figure 1b. Such a difference is related to the fact that the RI of ethanol and gasoline used in the simulations are not necessarily the same for AE and G87 gasoline. Nevertheless, the peak wavelength separation between AE and G87 gasoline should be enough to monitor the different gasohol blends.

Gasohol measurements were performed by first mixing the gasohol blends for a period of 2 min. After mixing, the gasohol was inserted into the channel and the spectral response of the MMI sensor was acquired with the OSA. Before a new measurement the MMI sensor is rinsed with pure ethanol and, after filling the channel with a new gasohol blend, the spectral response is measured again. The spectral response of the MMI sensor for each one of the gasohol blends, as listed in Table 1, is shown in Figure 4b. We can observe that the spectral response of the MMI sensor is shifted to longer wavelengths as the amount of AE is reduced from the gasohol blend. Such response is correlated with the fact that the G87 gasoline has a higher RI than the AE.

**Figure 5.**
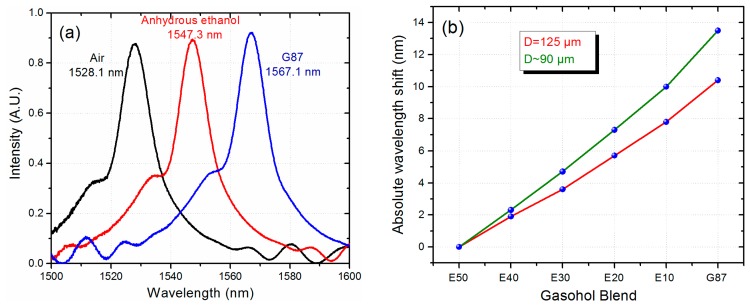
(**a**) Spectral response of the MMI sensor for AE and G87 gasoline (No-Core MMF diameter of 90 μm); (**b)** Absolute peak wavelength shift of both MMI sensors as a function of the gasohol blends.

As shown in Figure 4b, the sensor can clearly identify the different gasohol blends. However, a simple way to slightly enhance the sensitivity of the MMI sensor is by reducing the diameter of No-Core fiber, which effectively increases the interaction between the evanescent field and the gasohol. Using a buffered oxide etching (BOE) solution, which is a mixture of ammonium fluoride and hydrofluoric acid (6:1 volume ratio), the external diameter of the No-Core fiber (originally 125 μm) was reduced to approximately 90 μm by applying an etching time of 130 min. In order to achieve a specific peak wavelength after the etching, the length of the No-Core fiber is calculated using Equation (1) with the target diameter of 90 μm. In this particular case the length of the No-Core fiber was 31.35 mm for a peak wavelength close to 1530 nm. We should highlight that, before etching, the transmitted spectra does not show any noticeable peak related to the image. As the fiber is being etched, we can observe a well-defined peak appearing from the long wavelength edge of the transmitted spectra. As the etching continues, the whole spectrum is shifted to shorter wavelengths until we reach the desired peak wavelength value. As shown in Figure 5a the peak wavelength is very close to the design peak wavelength of 1530 nm. Also shown in Figure 5a is the spectral response of the modified MMI sensor when is covered with AE and G87 gasoline. Here we observe an increment in the peak wavelength difference between AE and G87 gasoline of 19.8 nm. We measured the gasohol blends using this sensor and, as shown in Figure 5b, a sensitivity of 0.270 nm/%AE is obtained as compared to a sensitivity of 0.208 nm/%AE from the original MMI sensor without etching. We should also highlight that the response of both sensors are highly linear.

**Figure 6.**
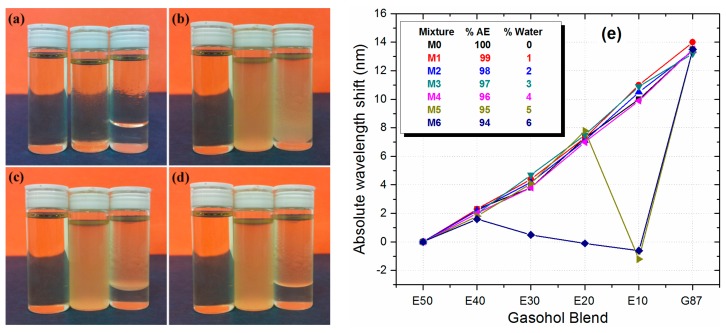
E10 gasohol samples with different water percentage of 1%, 5%, and 10% (left to right) with (**a**) After adding AE/water to G87; (**b**) After shaking the samples; (**c**) Three seconds after shaking; (**d**) Six seconds after shaking; and (**e**) Absolute peak wavelength response of the MMI sensor as function of gasohol blends with different water volumes.

A more critical issue when monitoring gasohol blends is related to the capability of ethanol to absorb water. As we previously described, there is a maximum amount of water that the blend can hold before phase separation issues occur. This limit is at 4% of water volume with respect to the ethanol volume that will be mixed with gasoline. This effect can be easily observed in Figure 6a–d. The gasohol blend in this case is E10 that corresponds to 90% of gasoline and 10% of AE. The bottles shown in each figure, going from left to right, have a water volume of 1%, 5%, and 10% with respect to the AE volume. Figure 6a shows the gasohol blend when the water/AE mixture is gently added to the G87 gasoline. We can notice that the bottle with 1% of water volume is well mixed with the gasoline, whilst the one with 10% of water volume immediately exhibited a phase separation process. The bottle with 5% water volume does not experience a drastic phase separation process, but the liquids are not homogeneously mixed, instead a slightly cloudy colloidal suspension is obtained. Figure 6b–d, correspond to snapshots taken every three second after the bottles have been shaken for one minute. We can easily observe that the E10 blend with 1% water volume remains practically unaltered. However, the E10 blends with water volumes of 5% and 10% become cloudy due to the inability of the ternary constituents to be mixed. We can also notice that the blend with 10% water volume goes into phase separation very rapidly, while the 5% water volume takes a longer time on the order of 2 min.

We evaluated the ability of the etched MMI sensor (90 μm diameter) to detect water content in the different gasohol blends shown in Table 1. For each gasohol blend we prepared a set of samples with different water content from 0% to 6% and increments of 1% (labeled M0 to M6), with respect to the total AE volume. The gasohol blend-water mixture was vigorously shaken before every measurement, then it was introduced into the channel, and the transmitted spectrum is immediately acquired. As shown in Figure 6e, all gasohol blends with water content from 0% to 4% exhibit a similar response as before.

**Figure 7.**
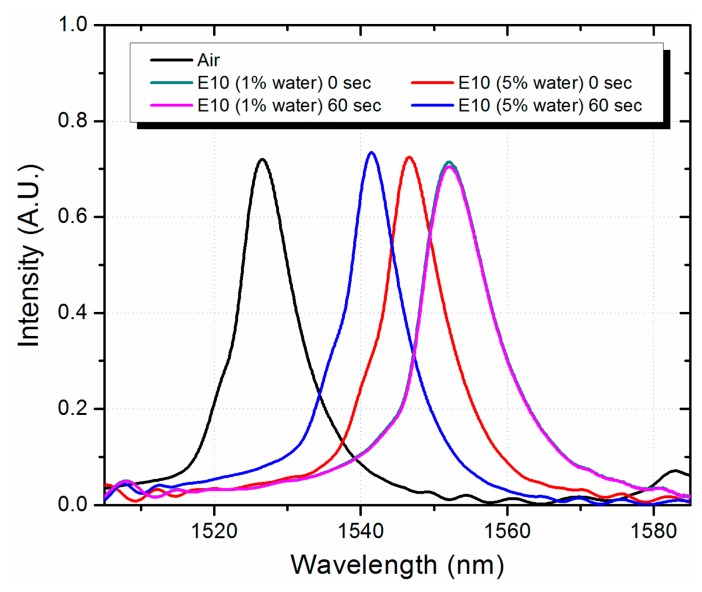
Spectral response of the MMI sensor for E10 gasohol with different water concentrations (1% and 5%) acquired right after shaking (0 s) and when the gasohol has been settled during 60 s.

We only notice a slight change in the sensitivity which can be related to the different water content. Nevertheless, when the water content increases to 5% we observe a significant deviation of more than 10 nm from the linear response for the E10 gasohol blend. We can also notice that the as the water content increases to 6%, a similar effect is observed for the E20 and E30 gasohol blends. This behavior is related with the formation of small droplets of gasoline and AE with water due to the phase separation, which effectively reduces the RI for the gasohol blend. This also reduces the effective RI and diameter of the fundamental mode in the MMI device, and the peak wavelength is also reduced. At higher water volume the effect is seen by all the gasohol blends. In fact, since complete phase separation occurs in a matter of seconds for high water volumes, in a real application the sensor can be placed close to the bottom of the gasohol container. In this scenario the RI seen by the MMI sensor under complete phase separation will be that of the AE with water, which should be very close to that of the AE, and a larger peak wavelength deviation from linearity should be observed. We performed such experiment by fixing the MMI sensor at the bottom of a container where we could add the gasohol blends. The container is large enough in order to have the MMI completely covered with the mixture of AE with water when phase separation occurs. As shown in Figure 7, for an E10 gasohol blend and 1% water content the MMI peak wavelength is the same right after shaking the sample and after one minute. In fact, we measured the spectrum after a couple of minutes and the MMI peak wavelength did not change.

However, when the water content is raised to 5%, the MMI peak wavelength right after shaking the sample is shifted by 5.4 nm to shorter wavelengths. After one minute, when phase separation is observed, the MMI peak wavelength shifts another 5.2 nm. This provides a peak wavelength difference of 10.6 nm as compared to the gasohol sample that does not experience phase separation. In this way we can clearly determine if the gasohol blend has exceeded or not the water limit of 4%. We should note that these experiments were performed using the No-Core fiber with a diameter of 125 μm. Therefore, a higher wavelength difference should be obtained by using a reduced core No-Core fiber. The results demonstrate the feasibility of employing the MMI sensor as a reliable system for gasohol quality control that is not only a simple but also a cost effective system.

## 5. Conclusions

A novel gasohol fuel detection system based on MMI fiber sensors was demonstrated. The MMI sensor relies on the fact that the RI of the gasohol blend is reduced as the ethanol content is increased. Since MMI sensors are capable of detecting small RI changes, accurate control of gasohol blends is achieved by tracking the peak spectral response of the MMI sensor. Gasohol blends with ethanol contents from 0% to 50% are clearly identified with a maximum sensitivity of 0.270 nm/%AE. The sensor is also capable of detecting when the water content of the gasohol blend exceeds the maximum volume that induces phase separation effects. When this occurs the liquids are not homogeneously mixed and a slightly cloudy colloidal suspension is obtained. Since the effective RI of the suspension is lower than the homogeneous mixture, we obtain a shorter peak wavelength response that the deviates from the linear response of the MMI sensor. Since the MMI sensor is straightforward to fabricate and does not require any special coating it is a cost effective solution for monitoring the quality of gasohol blends.

## Figures and Tables

**Table 1.** Gasohol blends prepared using anhydrous ethanol and G87 gasoline. The blends are labeled following standard convention.

**Solution**	**G87 (mL)**	**Anhydrous Ethanol (mL)**	**G87/AE (%)**
G87	10	0	100/0
E10	9	1	90/10
E20	8	2	80/20
E30	7	3	70/30
E40	6	4	60/40
E50	5	5	50/50
